# Dynamic Regimes of El Niño Southern Oscillation and Influenza Pandemic Timing

**DOI:** 10.3389/fpubh.2017.00301

**Published:** 2017-11-23

**Authors:** Olusegun Steven Ayodele Oluwole

**Affiliations:** ^1^Neurology Unit, College of Medicine, University of Ibadan, Ibadan, Nigeria

**Keywords:** El Niño, influenza, pandemic, dynamics, nonlinear, determinism, chaos, fractal

## Abstract

El Niño southern oscillation (ENSO) dynamics has been shown to drive seasonal influenza dynamics. Severe seasonal influenza epidemics and the 2009–2010 pandemic were coincident with chaotic regime of ENSO dynamics. ENSO dynamics from 1876 to 2016 were characterized to determine if influenza pandemics are coupled to chaotic regimes. Time-varying spectra of southern oscillation index (SOI) and sea surface temperature (SST) were compared. SOI and SST were decomposed to components using the algorithm of noise-assisted multivariate empirical mode decomposition. The components were Hilbert transformed to generate instantaneous amplitudes and phases. The trajectories and attractors of components were characterized in polar coordinates and state space. Influenza pandemics were mapped to dynamic regimes of SOI and SST joint recurrence of annual components. State space geometry of El Niños lagged by influenza pandemics were characterized and compared with other El Niños. Timescales of SOI and SST components ranged from sub-annual to multidecadal. The trajectories of SOI and SST components and the joint recurrence of annual components were dissipative toward chaotic attractors. Periodic, quasi-periodic, and chaotic regimes were present in the recurrence of trajectories, but chaos–chaos transitions dominated. Influenza pandemics occurred during chaotic regimes of significantly low transitivity dimension (*p* < 0.0001). El Niños lagged by influenza pandemics had distinct state space geometry (*p* < 0.0001). Chaotic dynamics explains the aperiodic timing, and varying duration and strength of El Niños. Coupling of all influenza pandemics of the past 140 years to chaotic regimes of low transitivity indicate that ENSO dynamics drives influenza pandemic dynamics. Forecasts models from ENSO dynamics should compliment surveillance for novel influenza viruses.

## 1 Introduction

Influenza epidemics occur annually during the winter of northern and southern hemispheres (1), but only five influenza pandemics occurred between 1899 and 2016 (2). Putative risk factors for influenza pandemics, which include school calendar, demography, geography, changes in virulence of influenza A viruses, and waning immunity (3), are, however, the same for seasonal influenza epidemics in these regions. In the tropics where humidity is relatively high all year, influenza epidemics peak once, twice, or are endemic (4). Seasonal influenza epidemics, however, peak during the rainy season in some tropical regions (5). Transmission of influenza viruses in human populations occurs through aerosols (6), droplets (7), or direct contact with infected secretions (8), but aerosol transmission is the most effective (9). While influenza viruses are present constantly in the air, seasonality of epidemics in the northern and southern hemispheres is linked to enhanced survival and transmission during low precipitation (10, 11). El Niño southern oscillation (ENSO), which modulates global precipitation, has been correlated with seasonality of influenza epidemics (1). Analysis of historical data from 1580 to 2013 showed that influenza pandemics lagged peaks of El Ninos by 0–2 years (12). Analysis of monthly ENSO time series, however, showed that influenza pandemics lagged El Ninos by 0–2 months (2). Influenza epidemics and pandemics, therefore, appear coupled to El Niños.

ENSO is coupled ocean–atmosphere system in equatorial Pacific Ocean. Southern oscillation, the atmospheric component of ENSO, is the alternating low and high sea level pressures between the west and east equatorial Pacific Ocean (13), while sea surface temperature, the oceanic component of ENSO, is the alternating warm and cool sea surface between the west and east equatorial Pacific Ocean (14). The phase of ENSO is neutral when easterly winds move warm sea surface water from east to west equatorial Pacific (14), where warm and moist air rises, condenses, and gives rain. The wind blows eastwards in the upper atmosphere, becomes dry and cool, and sinks in east Pacific Ocean to complete the Walker circulation (14). ENSO is in El Niño phase, which occurs irregularly about 2–7 years, when the easterly winds weaken and warm sea surface water move from equatorial west to east Pacific. La Niña phase of ENSO occurs when very strong easterly winds move surface water westward and make the sea level temperature of the east Pacific abnormally cool (15). Warming of equatorial east Pacific ocean about Christmas is an annual event that has been observed for over a century (16, 17). Currently, however, El Niño is defined as sea surface temperature anomalies in equatorial east Pacific Ocean ≥0.5°C, which lasts five consecutive overlapping 3-month periods in the Niño 3.4 region (5°N–5°S 120°–170°W) (18), while La Niña is defined as cooling of ≥−0.5°C for similar period. Unlike the annual warming events, the frequency and strength of El Niños vary on decadal and multidecadal timescales (19).

ENSO time series is composed of nonlinear components with timescales from sub-annual to multidecadal (20, 21). The component of ENSO that modulates climate on annual timescale (22) is phase-locked to season cycles such as El Niño (23) which typically starts in spring and peaks in winter. The dynamics of ENSO annual component has been shown to drive the dynamics of seasonal influenza (21), but while severe seasonal influenza epidemics and the 2009–2010 pandemic occurred during chaotic regime of ENSO dynamics, it is not known if this is true for all influenza pandemics. ENSO dynamics from 1876 to 2016 were characterized to determine if influenza pandemics are coupled to chaotic regimes.

## 2 Materials and Methods

Monthly time series of southern oscillation index (SOI) from 1876 to 2016 was obtained from ftp://ftp.bom.gov.au/anon/home/ncc/www/sco/soi/soiplaintext.html. SOI is derived from the difference of surface air pressure of Tahiti and Darwin, Australia. Niño3.4 sea surface temperature (SST) was obtained from https://climexp.knmi.nl/data/iersst_nino3.4a.dat. Niño3.4 region spans latitude 5N–5S and longitude 170–120 W. Historical records of El Niño were obtained from https://sites.google.com/site/medievalwarmperiod/Home/historic-el-nino-events and http://www.bom.gov.au/climate/enso/enlist/, and of La Niña from http://www.bom.gov.au/climate/enso/lnlist/index.shtml. Further records were from Ref. (24) and Oceanic Niño index (25). Historical records of onsets and peaks of influenza pandemic waves from 1876 to 2016 were obtained from the literature (3, 26–30).

### 2.1 Spectra and Oscillatory Components of SOI and SST

Presence of nonlinearities in time series of southern oscillation and sea surface temperature were determined using the third order moment method (31). The highest p-value for rejecting the null hypothesis of linearity and stationarity was <0.001. The synchrosqueeze transform algorithm (32) was applied to each time series to compute the time-varying spectra.

Linear and nonlinear dynamic systems are typically composed of multiple components with different timescales (33). While linear dynamic systems are composed of time-invariant components, nonlinear dynamic systems are composed of time-varying components. Monocomponent nonlinear dynamic system without noise can be modeled as Ψ(*t*) = *r*(*t*)*cos*[2*πϕ*(*t*)] (33), where *r*(*t*) is time-varying amplitudes and *ϕ(t)* is time-varying phases. Multicomponent nonlinear dynamic system without noise, however, can be modeled as the sum of its components $\Psi (t)=\sum_{k=1}^{k}\;r(k)cos[2\pi \phi (k)]$. Multicomponent dynamic system must, therefore, be decomposed to components before meaningful instantaneous amplitudes and phases can be determined. The empirical mode decomposition (EMD) algorithm (34) filters nonlinear dynamic systems to intrinsic mode functions (IMFs) or modes, which are components that can be represented as *ψ*(*t*) = *r*(*t*)*cos*[2*πϕ*(*t*)] where the amplitude and phase are physically meaningful. The components can be summed to regenerate the original dynamic system. Noise-assisted multivariate empirical mode decomposition algorithm (35) was used to decompose SOI and SST to components. The algorithm of multivariate EMD (35) is as follows:

1. Generate pointset based on Hammersley sequence for sampling on an (*n* − 1) – *sphere*.
2. Calculate projection ${\left\{p^{\theta_{k}}(t)\right\}}_{t=1}^{T}$, of the input signal ${\left\{v(t)\right\}}_{t=1}^{T}$ along the direction of vector ${\text{x}}^{\theta_{k}}$, for all *k* (the whole set of direction vectors), giving ${\left\{p^{\theta_{k}}(t)\right\}}_{k=1}^{K}$ as the set of projections.
3. Find the time instants ${\left\{t^{\theta_{k}}\right\}}_{k=1}^{K}$, which correspond to the maxima of projected signals set.
4. Interpolate $\left[t^{\theta_{k}},\text{v}(t^{\theta_{k}})\right]$ for all values of *k*, to obtain multivariate envelope curves ${\left\{{\text{e}}^{\theta_{k}}(t)\right\}}_{k=1}^{K}$.
5. For a set of *K* direction vectors, calculate mean **m**(*t*) of the envelope curves as
  $$\text{m}(t\text{)}=\frac{1}{K}\sum_{k=1}^{K}\;e^{\theta_{k}}(t).$$
6. Extract detail *d*(*t*) using *d*(*t*) = *x*(*t*) − *m*(*t*). If the detail *d*(*t*) fulfils the stoppage criterion for multivariate intrinsic mode function (IMF), apply the above procedure to *d*(*t*), otherwise apply it to *x*(*t*) − d(*t*).

The components of SOI and SST were fast Fourier transformed to determine the timescales.

### 2.2 Trajectories and Attractors

Each component was Hilbert transformed to analytical signal Ψ(*t*) = *r*(*t*)*exp*[*iθ*(*t*)], which has real and imaginary components. The time-varying magnitude of the analytical signal is its absolute value, which is also called envelope or amplitude, while the time-varying phase is the argument of the analytical signal.

The state, phase, or vector space describes the time-varying states of dynamic systems in multidimensional space. The state space of dynamic systems such as the logistic map or Lorenz system are usually modeled with differential equations, which unfortunately are not available for most dynamic systems. When differential equations are unavailable, however, multidimensional state space can be reconstructed by transforming unidimensional scalar time series {*x_i_, i* = 1,2,…} of the dynamic system to delay vector coordinates {*X*_i_ = *x*_i_, *x_i+L_, x_i+2L_*,…,*x_i+_*_(_*_m–1_*_)_*_L_*}, where *m* is embedding dimension, and *L* is delay or lag (36). This reconstruction, which is based on the Taken’s embedding theory (37), recovers the topology of dynamic systems from delay vector coordinates. The state spaces of SOI and SST components were reconstructed using delayed embedding vector coordinates (37). The embedding dimensions *m* were calculated using Cao’s algorithm (38), and lags *τ* were estimated using mutual information algorithm (39).

Fractals are geometrical objects with self-similarity and long memory properties. The Hurst exponent is a metric of fractality that is defined as *x*(*_t_*) = *a^H^x*(*at*). The Hurst exponent is time-invariant in mathematical fractals, but time-varying in statistical fractals (40), which are described as multifractals (41, 42). Multifractal detrended fluctuation analysis (42) of SOI and SST components was performed to determine if the Hurst exponents are time-varying (42). Fractal dimension is an indicator of chaos, which is characterized by exponential divergence of trajectories that have minimally different initial conditions. The Lyapunov exponent (λ), which measures the average rate of divergence of close trajectories (43), is a metric of chaos that is expressed in the relation $\frac{d}{d_{0}}=\exp^{\lambda (t-t_{0})}$, where *d*_0_ is small displacement from initial position at time *t*_0_, and *d* is displacement at time *t* > *t*_0_. When λ > 0, $\frac{d}{d_{0}}$ grows exponentially and the dynamics becomes chaotic (43), but λ < 0 indicates decay to steady state. The Lyapunov spectra were constructed for each component to determine sensitivity to initial conditions.

Recurrence plots of SOI and SST components were generated to characterize dynamic regimes. Recurrence plot is the graphical display of recurrence in two dimensions (44), which characterizes the presence of regimes, transitions, coupling, and synchronization of dynamic systems in state space (45). Since trajectories do not return to exact regions of state space ${\vec{x}}_{i}{\vec{x}}_{j}$, the neighborhood that trajectories return is defined by the threshold *ϵ*(45). The recurrence matrix is defined as $R_{i,j}(\varepsilon )=\;\Theta \left(\varepsilon -\left\|{\vec{x}}_{I}-{\vec{x}}_{j}\right\|\right)$, *i, j* = 1,…, *N*, where *N* is the number of measured points ${\vec{x}}_{i}$, *ϵ* is the threshold distance, Θ(.) is the Heaviside function, and ||⋅|| is the norm (45). When a trajectory in state space $\left({{\vec{x}}_{i}}_{i=1}^{N}\right)$ visits a state ${\vec{x}}_{i}\approx {\vec{x}}_{{\;}_{j}}$ the recurrence **R***_i,j_* is 1, but when the trajectory visits a state x→i≈x→ j the recurrence **R***_i,j_* is 0. The typology of recurrence plot is the large-scale structure, which has homogeneous pattern for uniformly distributed white noise, but has long uninterrupted checkerboard lines for periodic or quasi-periodic systems (45). The texture of recurrence plot is the small-scale structure, which includes single dots, and lines that can be diagonal, vertical, or horizontal (45). Periodic dynamic systems have long, uninterrupted, even parallel diagonal lines, but quasi-periodic dynamic systems have uneven vertical distances between the diagonal lines. Chaotic dynamic systems, however, have single points, short diagonal lines, as well as vertical and horizontal lines (46).

Metrics of recurrence quantification analysis were calculated for 36-month windows of the recurrence matrix along the line of identity to determine the transitions of dynamic regimes (47). Determinism (DET) and divergence (DIV) were derived from diagonal line structures of recurrence plots, while laminarity (LAM) and trapping time (TT) were derived from vertical line structures of recurrence plots (45, 48). Determinism is the ratio of recurrence points that form diagonal lines of at least *l_min_* and all recurrence points, and divergence is the inverse of the longest diagonal line *L_max_* (47). Laminarity is the ratio of recurrence points that form vertical lines of at least *l_min_* and all recurrence points, while trapping time is the average length of vertical structures, which estimates the time duration the system is in specific state. To determine the statistical significance of the metrics, 5,000 bootstrap samples of the values of the metrics were compared with 5,000 bootstrap samples of the metrics determined for random time series.

### 2.3 Joint Recurrence and Peaks of Influenza Pandemics

Since ENSO is coupled to season cycles, the typology and texture of SOI and SST joint recurrence of annual components were characterized to determine the dynamic regimes when influenza pandemics occurred. Each pandemic period was defined from 18 months before the first peak through to 18 months following the first peak. This created 36-month window for each pandemic in the dynamics of ENSO, which included the multiple peaks of each pandemic.

Joint recurrence plot assesses the probability that similar points in state space are visited by two chaotic dynamics (45). The cross correlation index (CPR) was calculated for 36-month windows of joint recurrence plot to determine if the trajectories of SOI and SST annual components are synchronized. The CPR index was calculated by comparing the probability *P*(*ϵ*)(*τ*) that the trajectory returns to *ϵ*-neighborhood of a previous point on the trajectory. The cross correlation between *P*_1_(*τ*) and *P*_2_(*τ*) is defined as $CPR=\langle {\bar{P}}_{1}(\tau )P_{2}(\tau )\rangle /(\sigma_{1}\sigma_{2})$. When the trajectories are in phase synchrony, the probability of recurrence is maximal at the same time, and *CPR* ≈ 1.

Complex network is characterized completely by the adjacency matrix, which can be obtained by transforming the recurrence matrix. The vertices of recurrence complex network represent state vectors, while the edges represent proximity between vertices (49). Complex network was generated from adjacency matrix of SOI and SST joint recurrence of annual components. Degree centrality and its distributions were calculated for networks and sub-networks of El Niño and La Niña. Distribution of degree centrality of El Niños lagged by influenza pandemics was compared with other El Niños, and with very strong, strong, moderate, and weak El Niños. Bootstrap statistics was used to compare metrics with random time series.

Metrics of network analysis, which maps time series to complex networks with distinct topological characteristics (50), have been used to detect regimes and transitions of dynamic systems. These metrics are computed from adjacency matrix, which has correspondence to the recurrence matrix. Transitivity dimension, which is defined as $D_{\text{T}}(\epsilon )=\frac{log\text{T}(\epsilon )}{log(3/4)}$ (51), was calculated for 36-month windows of SOI and SST joint recurrence of annual components. To determine statistical significance, 5,000 bootstrap samples of values of transitivity dimensions were compared with 5,000 bootstrap samples of transitivity dimensions of random time series.

### 2.4 Scripts, Programming, and Statistical Packages

Matlab scripts of relevant algorithms were used to test for nonlinearities in time series (31), noise-assisted multivariate empirical mode decomposition (52), and multifractal detrended fluctuation analysis (42). Matlab scripts were implemented in Matlab-Octave programming language.

Lyapunov spectrum, lag, embedding dimension, Takens, and state space plots were implemented in the nonlinearTseries package of R Statistical Programming and Environment, Austria, version 3.2.2, 2015 (53). Embedding dimensions *m* were calculated for IMFs using Cao’s algorithm (38), and lags *τ* were estimated using mutual information algorithm (39). Pyunicorn package of Python programming language was used to generate matrices of recurrence plots and networks, the astropy package was used for bootstrap statistics, while the seaborn and matplotlib packages were used for graphics. Calculations of CPR index to determine synchronization of dynamics were performed as described in publication (54).

## 3 Results

**Figure 1:**
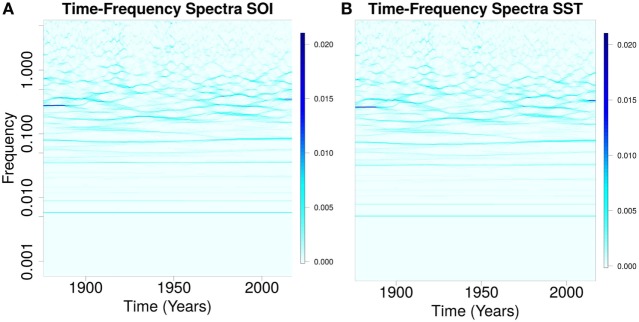
Spectra of southern oscillation (SOI) and sea surface temperature (SST). **(A)** (SOI) and **(B)** (SST) show similar oscillations with timescales from sub-annual to multidecadal.

**Figure 2:**
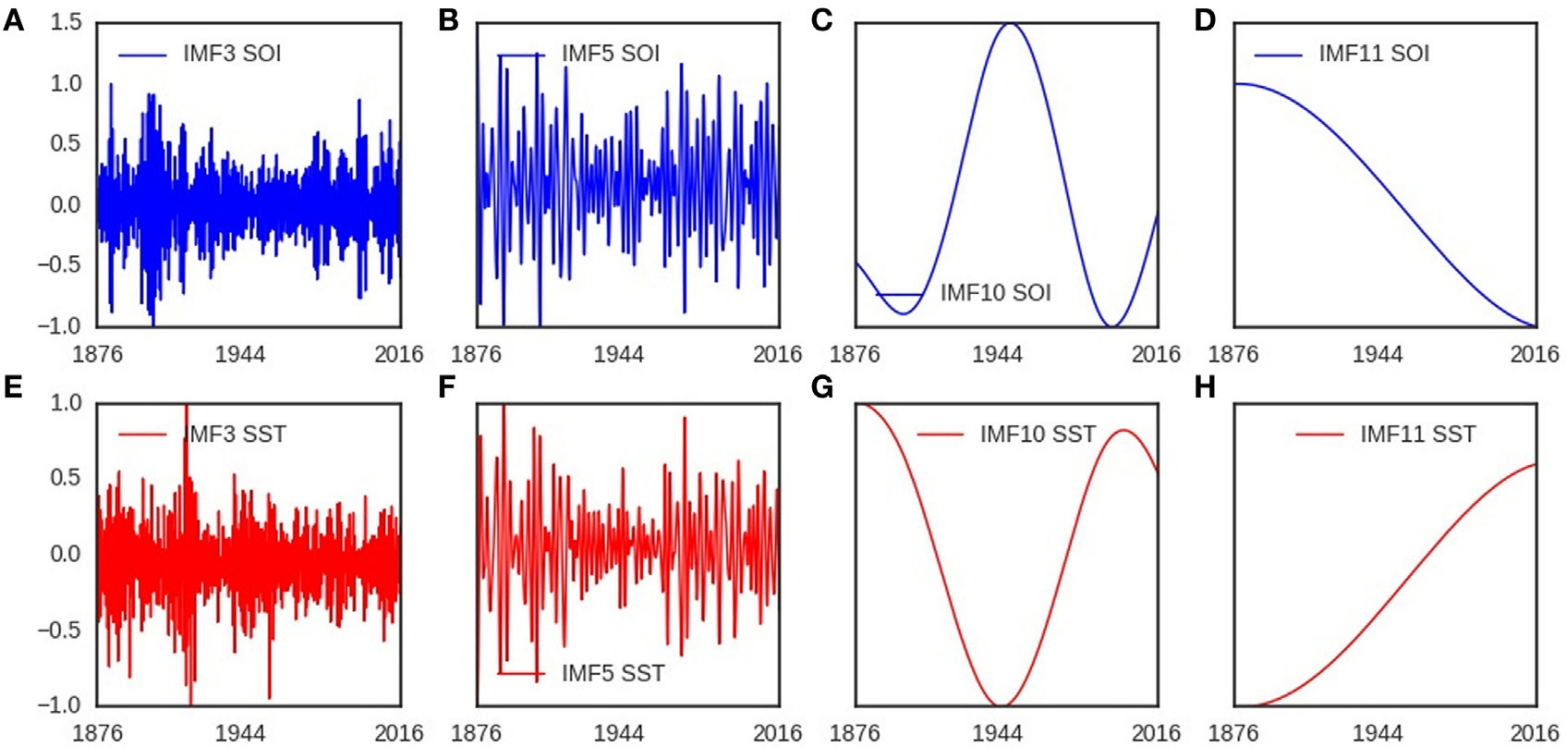
Monocomponents of southern oscillation (SOI) and sea surface temperature (SST). **(A–D)** show intrinsic mode functions of southern oscillation, which are the oscillatory components with different time scales. **(E–H)** show intrinsic mode functions of sea surface temperature, which are the oscillatory components with different time scales. The timescales of IMF_3_ is one year, but 172 years for IMF_10_.

The components of southern oscillation (SOI) and sea surface temperature (SST) anomalies showed similar time-varying spectra (Figures 1A,B). The timescales of these components ranged from sub-annual to multidecadal (Figures 1A,B). Eleven components (IMFs) were present in the noise-assisted multivariate empirical mode decomposition of SOI and SST. IMF_3,5,10,11_ for SOI is shown in Figures 2A–D, but in Figures 2E–H for SST. The trend of SOI anomalies troughed in 2016, but peaked for SST (Figures 2D,H). The timescales of SOI and SST components were 6 months for IMF_2_, 12 months for IMF_3_, 18 months for IMF_4_, 3.6 years for IMF_5_, 6.0 years for IMF_6_, 11.5 years for IMF_7_, 22.0 years for IMF_8_, 50.0 years for IMF_9_, and 172.0 years for IMF_10_.

### 3.1 Trajectories of Oscillatory Components

**Figure 3:**
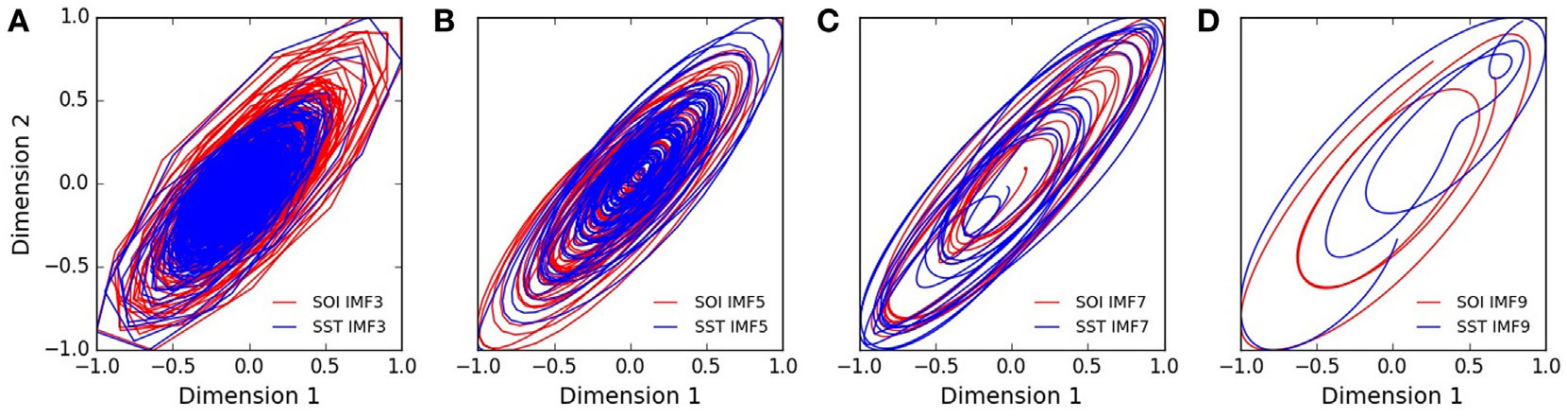
Trajectories of southern oscillation (SOI), and sea surface temperature (SST) in phase plane. **(A–D)** show the trajectories of intrinsic mode functions with different timescales. The geometries of the trajectories of SOI and SST are similar in state space.

In polar coordinates the trajectories of SOI and SST components show bounded spirals which decayed toward the origin. The trajectories of IMF_3,5,7,9_ in polar coordinates are shown in Figures S1A–D in Supplementary Material for SOI, but in Figures S1E–H in Supplementary Material for SST. In reconstructed state spaces, the trajectories of IMF_3,5,7,9_ are shown in Figures 3A–D for SOI and SST. The elliptical and bounded trajectories in reconstructed state spaces dissipated toward attractors. Visually the trajectories of SOI and SST components have similar geometry (Figures 3A–D). Lyapunov spectra of SOI and SST components showed multiple positive exponents, while their multifractal detrended fluctuation analysis showed time-varying Hurst exponents.

**Figure 4:**
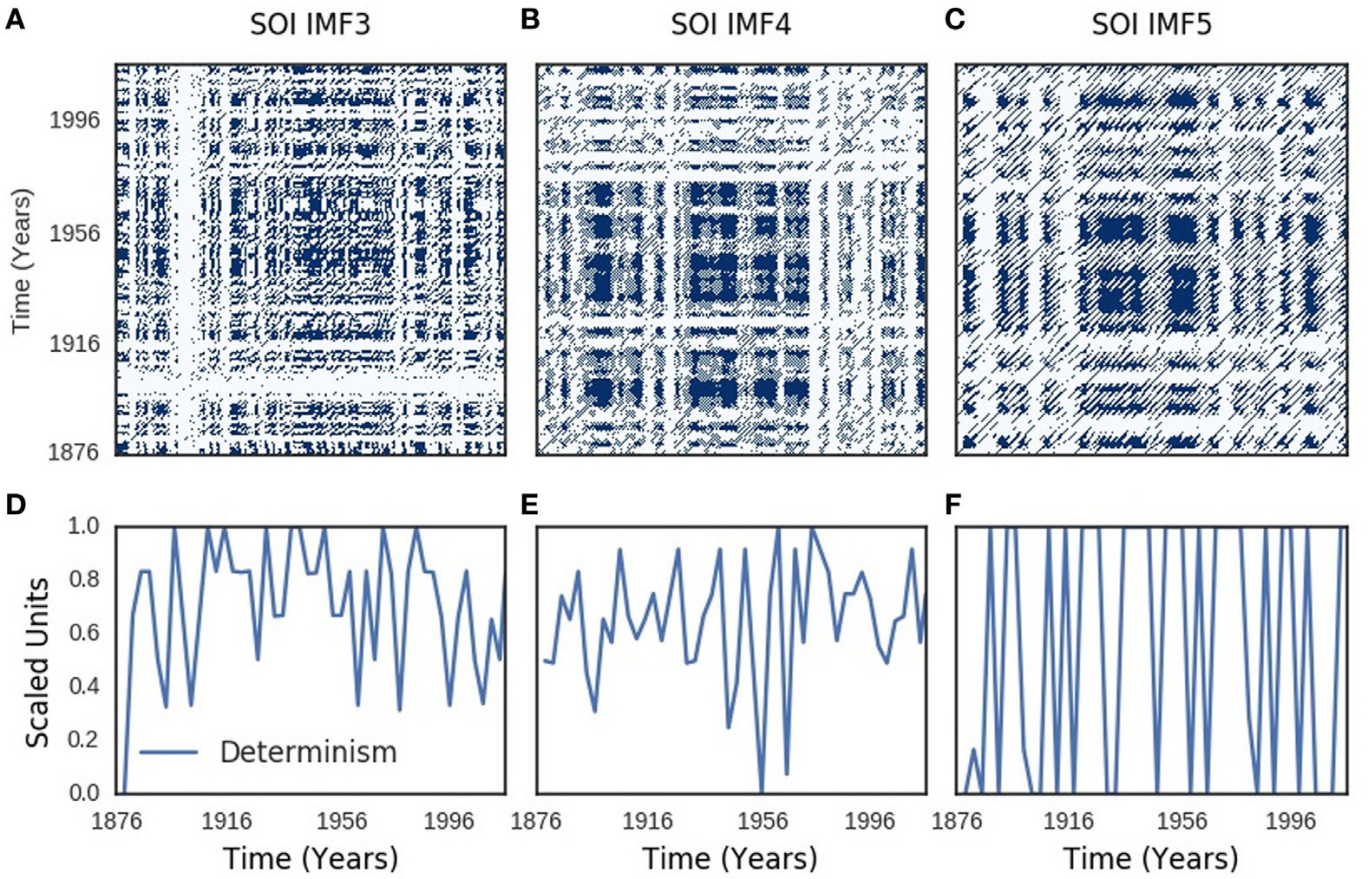
Southern oscillation (SOI) recurrence plots. Recurrence plots shown in **(A–C)** resemble Lorenz chaotic dynamics. Periodic regimes have long, uninterrupted, even parallel diagonal lines, while quasi-periodic regimes have uneven vertical distances between the diagonal lines. Chaotic regimes have single points and short diagonal lines. Time-varying determinism is shown in **(D–F)**.

**Figure 5:**
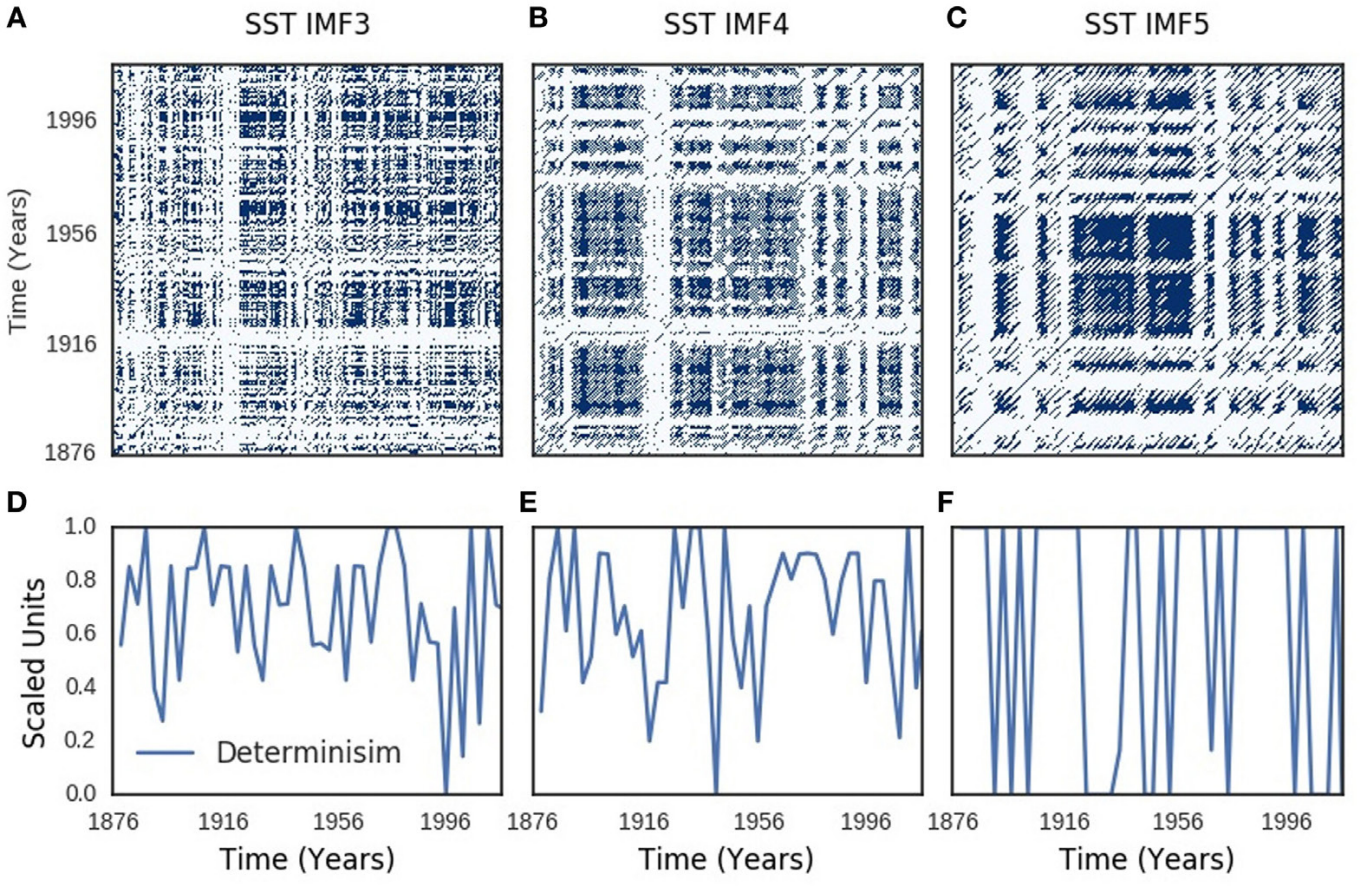
Sea surface temperature (SST) recurrence plots. Recurrence plots shown in **(A–C)** resemble Lorenz chaotic dynamics. Periodic regimes have long, uninterrupted, even parallel diagonal lines, while quasi-periodic regimes have uneven vertical distances between the diagonal lines. Chaotic regimes have single points and short diagonal lines. Time-varying determinism is shown in **(D–F)**.

Recurrence plots of SOI components showed periodic, quasi-periodic, and chaotic regimes (Figures 4A–C). Similar regimes were present in the recurrence plots of SST components (Figures 5A–C). Chaotic regimes, however, predominated in both recurrence plots. Geometrical mean (range) of time-dependent metrics of recurrence quantification analysis of SOI and SST were similar: 0.98 (0.93–0.99) for determinism of SOI, but 0.97 (0.91–1.00) for SST; 26 (13–40) for divergence of SOI, but 24 (12–42) for SST; 0.82 (0.61–0.93) for laminarity of SOI, but 0.84 (0.70–0.94) for SST; 3.0 (2.3–5.7) for trapping time of SOI, but 3.3 (2.3–6.6) for SST. Time-dependent plots of determinism, divergence, laminarity, and trapping time for SOI and SST dynamics are shown in Figures S2 and S3 in Supplementary Material. Bootstraps comparisons of these metrics with random time series were significantly different at p < 0.0001 for determinism, divergence, laminarity, and trapping times of SOI and SST.

### 3.2 Joint Recurrence of Annual Oscillatory Components and Influenza Pandemic Timing

**Figure 6:**
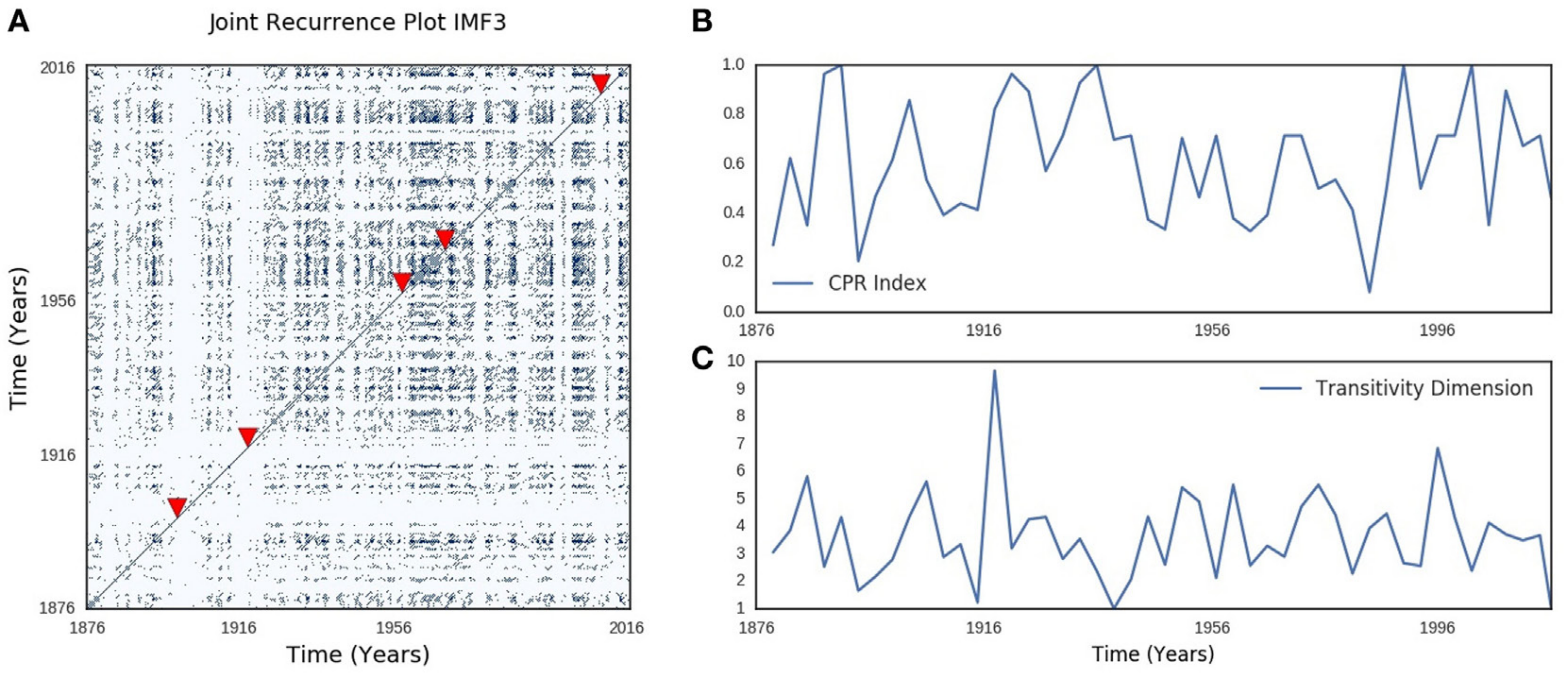
Southern oscillation (SOI) and sea surface temperature (SST) joint recurrence. **(A)** shows periodic, quasi-periodic, and chaotic regimes similar to the Lorenz model in the joint recurrence of annual oscillatory components of SOI and SST. The cross-correlation index in **(B)** shows the time-varying changes in synchronization of SOI and SST, while **(C)** shows time-varying changes in transitivity. The arrow heads indicate the timing of the first peak of each influenza pandemics. Metrics were calculated for 36-month windows that spanned 18 months before and after the first peak.

**Figure 7:**
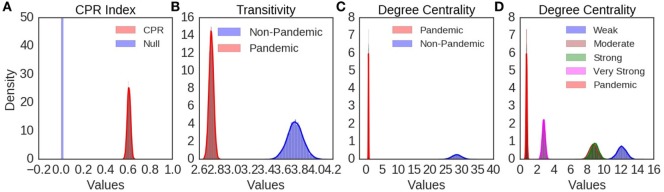
The distribution of bootstrap statistics is shown in **(A)** for cross correlation index (CPR), in **(B)** for transitivity, in **(C)** for degree centrality during El Ninos with pandemic and those without pandemic, and in **(D)** for degree centrality for El Ninos of different strength. The distribution of bootstrap statistics shows very highly significant statistical differences compared with random time series.

Joint recurrence plot of SOI and SST annual components showed similar typology and texture to their respective recurrence plots (Figure 6A). Periodic, quasi-periodic, and chaotic regimes were present in SOI and SST joint recurrence, but chaos–chaos transitions also dominate (Figure 6A). Five influenza pandemics from 1899 to 2016, which had precise records of months of onset and peak, were mapped to SOI and SST joint recurrence (Figure 6A). Influenza pandemics peaked in December 1899, December 1900, March 1901, March 1918, July 1918, November 1919, January 1920, October 1957, February 1958, March 1969, December 1969, January 1970, June 2009, and October 2009. All peaks of multiple waves of the influenza pandemics occurred during high divergence of SOI and SST trajectories. The first peak for each pandemic is shown in Figure 6A. The geometrical mean (range) was 0.6 (0–1) for cross correlation (CPR) index of SOI and SST joint recurrence of annual components (Figure 6B). Geometrical mean (range) of joint recurrence transitivity dimension was 3.50 (range 1.23–9.67) for dynamic regimes without influenza pandemics, but 2.71 (range 2.17–3.34) for dynamic regimes when influenza pandemic occurred (Figure 6C). CPR index was highly significantly different from null (p < 0.0001, Figure 7A). Transitivity dimensions of dynamic regimes when influenza pandemics occurred was highly significantly lower than for dynamic regimes without influenza pandemics (p < 0.0001, Figure 7B).

Undirected SOI and SST joint recurrence network of annual components had 6,185 nodes, and 134,205 edges. The degree centrality of the network showed power law distribution (Figure S4A in Supplementary Material). Sub-networks of El Niño and La Niña phases of ENSO also showed power law distributions of degree centrality (Figures S4B,C in Supplementary Material). Geometrical mean (range) degree centrality for the whole joint recurrence network was 22 (0–550). The number of nodes (edges) for sub-networks were 353 (5,075) for El Niño, 332 (4,727) for El Niño not lagged by influenza pandemics, but 21 (8) for El Niño lagged by influenza pandemics. Geometrical mean (range) degree centrality for sub-networks was 0 (0–3) for El Niños lagged by influenza pandemics, but it was 5 (0–88) for El Niños not lagged by influenza pandemics. The degree centrality of sub-network of El Niños lagged by influenza pandemics was highly significantly lower than that of El Niños not lagged by influenza pandemics (p < 0.0001, Figure 7C). It is also highly significantly lower than very strong, strong, moderate, and weak El Niños (p < 0.0001, Figure 7D).

## 4 Discussion

Southern oscillation index (SOI) and sea surface temperature (SST) have components with similar timescales, which indicate that the atmospheric and oceanic components of El Niño southern oscillation (ENSO) have oscillations with similar timescales (Figures 1A–B). Components of paleoclimate ENSO data of the past 21,000 years had timescales which ranged from sub-annual to 64 years (55). Multivariate empirical mode decomposition (MEMD) indicates that SOI and SST components have timescales from sub-annual to multidecadal (Figures 2A–C and Figures 2E–G). The basis for presence of multiple timescales can be linked to differences in the dynamics of upper and lower layers of the atmosphere and the ocean, which are driven by thermal, pressure, and Coriolis forces (56). Solar fluxes generate warmer region in the equator where air rises and moves poleward in the upper atmosphere (56), where wind speed is faster due to lower friction. The Coriolis force, which is generated by west to east rotation of the earth, however, deflects wind in the upper atmosphere to the right (eastward) in the Northern hemisphere, but to the left (westward) in the Southern hemisphere. Cold dense air at higher latitudes generates area of high pressure, which move in the lower atmosphere toward low pressure area of the equator (56). Wind stress or friction, which is the force per unit area exerted by the wind on the ocean, drives the dynamics of upper ocean layer. Satellite data from 1979 to 2013 show, however, that the dynamics of the atmosphere is not constant (57). The trend of SST which peaked during the 2014–2016 El Niño from its lowest value in 1876, and of SOI which troughed during the 2014–2016 El Niño from its peak in 1876 indicate multidecadal climate cycle during the period (Figures 2D,H). Occurrence of very strong El Niño in 1876, which caused severe droughts and floods (58, 59), and another very strong El Niño of 2014–2016, which is also causing severe droughts and floods, indicate determinism in the dynamics of ENSO. Since the very strong El Niños of 1982–1983 and 1997–1998 were followed by weaker ones, there is the likelihood that weaker El Niños will follow the 2014–2016 El Niño in the next decade. Thus, the timescales of atmospheric and oceanic components of ENSO provide spectra evidence of their coupling, while the trends of SOI and SST indicate that the very strong 2014–2016 El Niño occurred at the peak of multidecadal climate cycle.

### 4.1 Dynamics of Atmospheric and Oceanic Components of ENSO

The trajectory is the curve that describes the path of a signal as it evolves in time (60). In polar coordinates, the trajectory of a signal *x*(*t*) can be plotted in two-dimensional space as its instantaneous amplitude against phase ([*a*(*t*), *ϕ*(*t*)]). Theoretical ENSO models predict dissipative trajectory that is sustained by seasonal forcing (61, 62). External forcing supplies energy that sustains oscillation of dissipative dynamic systems. In polar coordinates, the trajectories of SOI and SST components, which show bounded spirals that grow and decay, indicate dissipative dynamics (Figures S1A–H in Supplementary Material). The trajectories of SOI and SST components in state space, which describes the time-varying states of dynamic systems in multidimensional space (36), also indicate dissipative dynamics (Figures 3A–D). The state space volume of dissipative dynamic systems contract and asymptotically approach a limiting value called the attractor as time *t*→∞ (63). The basin of attraction, however, is the set of initial points that determines the long-term behavior of trajectories. The varying divergence of trajectories of SOI and SST components that grow and decay toward a region of state space indicate that attractors and basins of attraction are present in the dynamics (Figures 3A–D). Thus, the dynamics of atmospheric and oceanic components of ENSO is dissipative.

Attractors of dissipative dynamic system’s may have simple or fractal geometry. The geometry of simple attractors, which has integer dimensions, includes the point in simple harmonic motion, the closed cycle in periodic dynamics, and the torus in quasi-periodic systems (60). Chaotic attractors, which are fractals, however, have geometry with non-integer dimensions (64). Sea surface temperature (SST) of east Pacific Ocean (62) and southern oscillation index (SOI) (65) have been shown to be fractals. Multivariate ENSO data from 2000 to 2015 have, however, been shown to be multifractal (21). The time-varying Hurst exponents indicate that SOI and SST components are multifractals. Fractal and multifractal geometry, however, indicate chaos, which is characterized by exponential divergence of trajectories that have minimally different initial conditions. The trajectories of SOI and SST components, which have positive Lyapunov exponents, therefore, indicate sensitivity to initial conditions. Thus, the dynamics of atmospheric and oceanic components of ENSO is chaotic.

Recurrence describes the return of trajectories of dynamic systems to the neighborhood of their initial states as time *t*→∞. The recurrence plots of SOI and SST trajectories show periodic, quasi-periodic, and chaotic regimes that resemble the recurrence of chaotic Lorenz system (45, 66), which models convective flow of the atmosphere (67) (Figures 4A–C and 5A–C). Chaos or turbulence occurs in fluids when inertial forces are dominant, but flow is laminar when viscous forces are dominant. Transition to chaos occur at certain parameters of motion (63), which in Ruelle–Takens route consists of three to four bifurcations from steady → periodic → quasi-periodic → turbulence or chaotic regimes(64). Chaos, however, also transits to chaos (68). Chaotic and quasi-periodic regimes, which were predicted by theoretical ENSO models (62, 69), are present in SOI and SST recurrence (Figures 4A–C and 5A–C). Chaos–chaos transition is, however, dominant (Figures 4A–C and 5A–C). Determinism, divergence, laminarity, and trapping time, which quantitatively assess periodic-chaos and chaos-periodic transitions, confirm presence of dynamic regimes in SOI and SST (Figures 4D–F and 5D–F; Figures S2 and S3 in Supplementary Material). Highly significant statistical differences of these metrics compared with random time series indicate that the dynamic regimes are deterministic. Thus, the dynamics of atmospheric and oceanic components of ENSO transit to chaos from periodic, quasi-periodic, and chaotic regimes.

### 4.2 SOI and SST Joint Recurrence and Influenza Pandemic Timing

The trajectories of two chaotic systems synchronize when coupling or forcing adjust their phases or amplitudes to evolve in a common state space (70). Similarity of state space geometry of SOI and SST suggests that their trajectories evolve in a common state space (Figures 3A–D). Joint recurrence assesses the probability that similar points in state space are visited by two chaotic trajectories of physically different time series (45). The topology of SOI and SST joint recurrence plot of annual components, which resembles their respective recurrence plots, supports visual interpretation that ENSO components have similar state space geometry (Figure 6A). The cross-correlation index (CPR) is a metric of synchronization which compares the probability *P*(*ϵ*)(*τ*) that a trajectory returns to ε-neighborhood of a previous point. The high CPR index of SOI and SST joint recurrence of annual components at all dynamic regimes indicate strong synchronization of atmospheric and oceanic components of ENSO (Figure 6B). The highly significant statistical difference of CPR index compared with null value of zero indicates that synchronization is not random (Figure 7A). Thus, the dynamics of SOI and SST are coupled.

Complex network, which models the vertices and edges of graphs (71), has been developed to characterize dynamics in state space. The distribution of degree centrality, which is defined as the number of edges connected to vertices, is a common metric of complex network (71). Small world networks have exponential distribution of degree centrality (72), while scale-free networks have power law distribution (73). Power law distribution of degree centrality of SOI and SST joint recurrence network, therefore, indicates scale-free network (Figure S4A in Supplementary Material). Scale-free sub-networks of El Niño and La Niña phases of SOI and SST joint recurrence network is characteristic of multifractal time series (74) (Figures S4B,C in Supplementary Material). Time-dependent transitivity dimensions indicate that regimes and transitions are also present in SOI and SST joint recurrence (Figure 6C). Thus, chaotic regimes are also present in dynamics of coupled atmospheric and oceanic components of ENSO.

Although the time series of influenza is not available from 1876, occurrence of all influenza pandemics during chaotic regimes of SOI and SST joint recurrence of annual components indicates strong relationship of influenza pandemics and climate dynamics (Figure 6A). The topology of recurrence plots, which shows that influenza pandemics occur during dynamic regimes of high divergence, and the highly significantly lower transitivity dimensions of these regimes indicate strong coupling of influenza pandemics to chaotic regimes (Figures 6A and 7B). Occurrence of influenza pandemics is, therefore, deterministic rather than random. El Niños that were lagged by influenza pandemics have been shown to have similar waveforms (2). The significantly lower degree centrality of sub-networks of El Niños that were lagged by influenza pandemics compared with other El Niños, and with El Niños of different strengths, indicate that influenza pandemics lag El Niños of distinct state space geometry (Figures 7C,D). Thus, climate dynamics determines the timing of influenza pandemics.

### 4.3 Emergence of Novel Influenza Viruses and Influenza Pandemic Timing

Influenza A viruses are ubiquitous in humans, birds, pigs, and several other mammals (75), but they do switch hosts to form novel strains (76). They are members of *Orthomyxoviridae* family, which have negative, single-stranded RNA. Novel influenza viruses, which cause influenza pandemics, arise when either of the two antigenic structural proteins, hemagglutinin (HA) and neuraminidase (NA) (77), undergo reassortment termed antigenic shift (75). Novel strains of influenza A viruses caused all five influenza pandemics which occurred between 1899 and 2016. These strains, however, circulated for years before the onset of pandemics (78). The triple reassortant influenza A(H1N1) 2009–2010, which contained gene segments from human, swine, and avian influenza A viruses (79, 80), circulated in swine in the 1990s (81), but human cases occurred about 5 years before 2009–2010 influenza pandemic (82, 83). The low aerosol transmission potential of novel reassortant influenza viruses (84), which increases following repeated mutations of HA and PB2 genes (85), is probably the basis for long circulation before onset of pandemics. Thus, timing of influenza pandemics is, therefore, not determined by emergence of novel influenza virus strains alone.

The guinea pig model showed that aerosol transmission of influenza viruses is dependent on temperature and precipitation (10). Low temperature and precipitation enhanced aerosol transmission of 2009–2010 influenza pandemic virus (86), whose isolates in northern France showed sensitivity to temperature (87). Spatiotemporal dynamics of influenza pandemics of 1889, 1957, and 2009 correlated with temperature in Sweden (30), while in Chile, a country that spans latitudes 17°S to 56°S that covers over 4,000 km, the north-south gradient of 2009–2010 influenza pandemic virus transmission correlated with low temperature and precipitation (88). Mortality from influenza in 359 USA counties from 1973 to 2002 correlated with <6 g of water vapor per kilogram of air (89). The varying impact of El Niño on global precipitation and temperature, which depends on strength and duration, explains while influenza pandemics do not lag all El Niños. Thus, decadal and multidecadal timing of influenza pandemics (2) is attributable to deterministic chaotic climate regimes which enhances global aerosol transmission of influenza viruses.

## 5 Conclusion

The timescales of SOI and SST components vary from sub-annual to multidecadal. The trends of SOI and SST anomalies that peaked during 2016 El Niño indicate that the strength of El Niños will decrease in the next few decades. The trajectories of SOI and SST components, and the joint recurrence of annual components are dissipative toward chaotic attractors. Chaos–chaos transitions dominate SOI and SST recurrence. SOI and SST joint recurrence of annual components show periodic, quasi-periodic, and chaotic regimes. All El Niños lagged by influenza pandemics have distinct state space geometry. Chaotic dynamics explains the aperiodic timing, and varying duration and strength of El Niños. Coupling of all influenza pandemics of the past 140 years to chaotic regimes of low transitivity indicate that ENSO dynamics drives influenza pandemic dynamics. It should, therefore, be feasible to developed predictive models of influenza pandemics from ENSO dynamics.

## Author Contributions

The author conceived, designed the methods, collected and analyzed data, and wrote the manuscript.

## Conflict of Interest Statement

The author declares that the research was conducted in the absence of any commercial or financial relationships that could be construed as a potential conflict of interest.

## Supplementary Material

Figure S1 Phase-amplitude plots. **(A–D)** show the phase-amplitude plots of intrinsic mode functions of southern oscillation (SOI). **(E–H)** show the phase-amplitude plots of intrinsic mode functions of sea surface temperature (SST).

Figure S2 Time-dependent recurrence quantification for southern oscillation. Plots of determinism, divergence, laminarity, and trapping time are compared.

Figure S3 Time-dependent recurrence quantification for sea surface temperature. Plots of determinism, divergence, laminarity, and trapping time are compared.

Figure S4 Powerlaw distributions of network degree centrality. Degree centrality of joint southern oscillation and sea surface temperature network is shown in **(A)**, of El Nino network in **(B)**, and of La Nina network in **(C)**.
