# Supplementary figures and images for: Dynamic Regimes of El Niño Southern Oscillation and Influenza Pandemic Timing
Source: Front Public Health. 2017 Nov 23;5:301. doi: 10.3389/fpubh.2017.00301 (PMC5703710; doi:10.3389/fpubh.2017.00301)

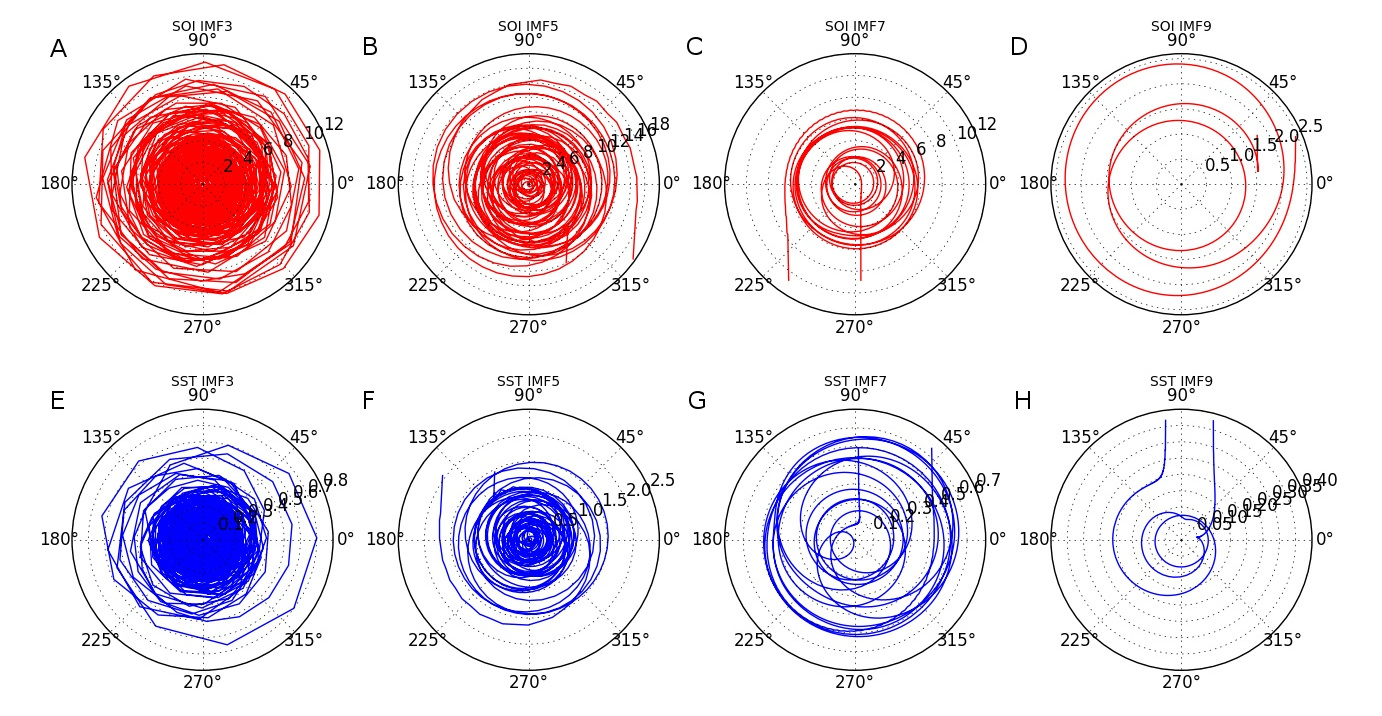

Supplement: Figure S1 — Phase-amplitude plots. (A–D) show the phase-amplitude plots of intrinsic mode functions of southern oscillation (SOI). (E–H) show the phase-amplitude plots of intrinsic mode functions of sea surface temperature (SST). [file image_1.jpeg]

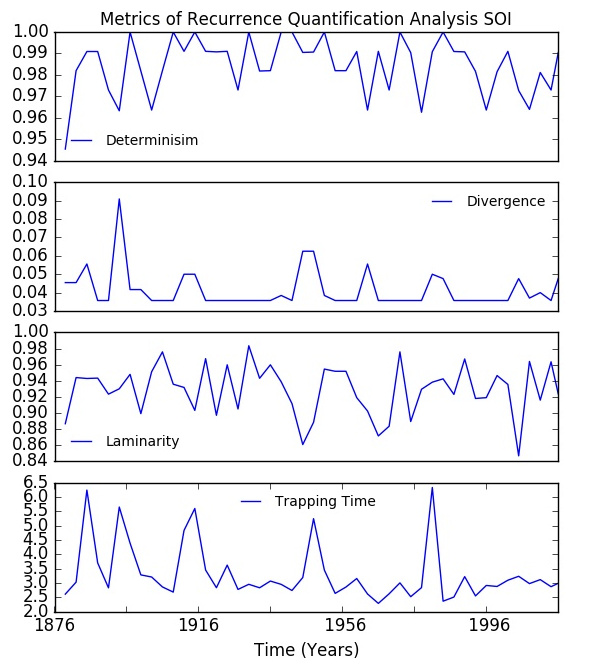

Supplement: Figure S2 — Time-dependent recurrence quantification for southern oscillation. Plots of determinism, divergence, laminarity, and trapping time are compared. [file image_2.jpeg]

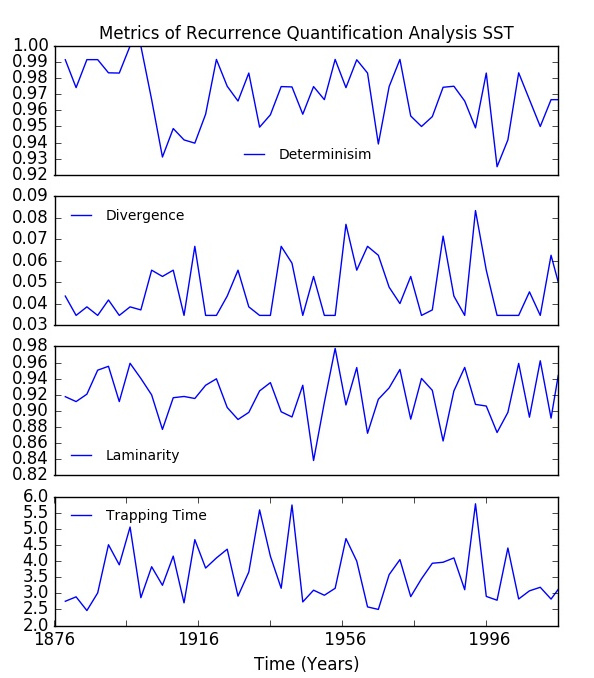

Supplement: Figure S3 — Time-dependent recurrence quantification for sea surface temperature. Plots of determinism, divergence, laminarity, and trapping time are compared. [file image_3.jpeg]

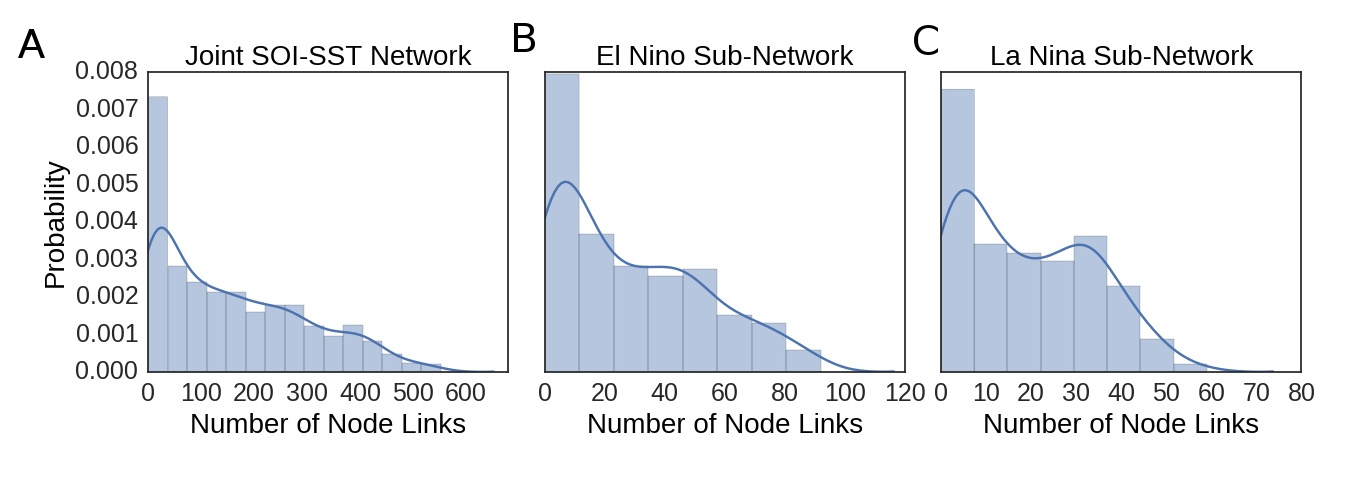

Supplement: Figure S4 — Powerlaw distributions of network degree centrality. Degree centrality of joint southern oscillation and sea surface temperature network is shown in (A), of El Nino network in (B), and of La Nina network in (C). [file image_4.jpeg]
